# The molecular mechanism for TERRA recruitment and annealing to telomeres

**DOI:** 10.1093/nar/gkae732

**Published:** 2024-08-27

**Authors:** Bersabel Wondimagegnhu, Wen Ma, Tapas Paul, Ting-Wei Liao, Chun Ying Lee, Samantha Sanford, Patricia L Opresko, Sua Myong

**Affiliations:** Program in Cell, Molecular, Developmental Biology and Biophysics, Johns Hopkins University, Baltimore, MD 21218, USA; Program in Cellular and Molecular Medicine, Boston Children's Hospital, Harvard Medical School, Boston, MA 02115, USA; Department of Physics, The University of Vermont, Burlington, VT 05405, USA; Program in Cellular and Molecular Medicine, Boston Children's Hospital, Harvard Medical School, Boston, MA 02115, USA; Program in Cellular and Molecular Medicine, Boston Children's Hospital, Harvard Medical School, Boston, MA 02115, USA; Department of Biophysics, Johns Hopkins University, Baltimore, MD 21218, USA; Program in Cellular and Molecular Medicine, Boston Children's Hospital, Harvard Medical School, Boston, MA 02115, USA; Department of Environmental and Occupational Health, University of Pittsburgh School of Public Health, Pittsburgh, PA 15261, USA; UPMC Hillman Cancer Center, Pittsburgh, PA 15213, USA; Department of Environmental and Occupational Health, University of Pittsburgh School of Public Health, Pittsburgh, PA 15261, USA; UPMC Hillman Cancer Center, Pittsburgh, PA 15213, USA; Program in Cell, Molecular, Developmental Biology and Biophysics, Johns Hopkins University, Baltimore, MD 21218, USA; Program in Cellular and Molecular Medicine, Boston Children's Hospital, Harvard Medical School, Boston, MA 02115, USA; Department of Biophysics, Johns Hopkins University, Baltimore, MD 21218, USA

## Abstract

Telomeric repeat containing RNA (TERRA) is a noncoding RNA that is transcribed from telomeres. Previous study showed that TERRA *trans* anneals by invading into the telomeric duplex to form an R-loop in mammalian cells. Here, we elucidate the molecular mechanism underlying TERRA recruitment and invasion into telomeres in the context of shelterin proteins, RAD51 and RNase H using single molecule (sm) assays. We demonstrate that TERRA *trans* annealing into telomeric DNA exhibits dynamic movement that is stabilized by TRF2. TERRA annealing to the telomeric duplex results in the formation of a stable triplex structure which differs from a conventional R-loop. We identified that the presence of a sub-telomeric DNA and a telomeric overhang in the form of a G-quadruplex significantly enhances TERRA annealing to telomeric duplex. We also demonstrate that RAD51-TERRA complex invades telomere duplex more efficiently than TERRA alone. Additionally, TRF2 increases TERRA affinity to telomeric duplex and protects it from RNase H digestion. In contrast, TRF1 represses TERRA annealing to telomeric duplex and fails to provide protection against RNase H digestion. Our findings provide an in-depth molecular mechanism underpinning TERRA recruitment and annealing to the telomere.

## Graphical Abstract

**Graphical Abstract:**
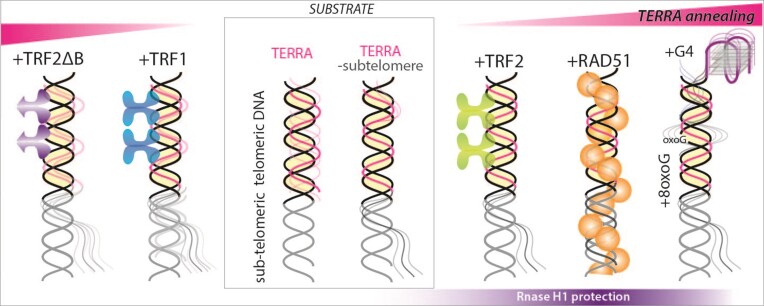

## Introduction

Telomeres are specialized DNA–protein complexes that cap the ends of linear chromosomes. Mammalian telomeres are composed of highly conserved tandem repeat DNA sequences of duplexed (TTAGGG)_n_, ending with a 3′ single stranded (TTAGGG)_n_ sequence, that is bound to telomere-specific proteins known as the shelterin complex. The shelterin complex, composed of TRF1, TRF2, POT1, TIN2, TPP1 and RAP1 proteins, plays a crucial role in maintaining telomere integrity through its association with telomeric DNA (1–3). This complex not only protects chromosome ends from degradation by nucleases but also prevents the improper activation of DNA damage response pathways (4,5).

TERRA (Telomeric Repeat-containing RNA) is a non-coding RNA transcribed by polymerase II using the C-rich strand of telomeres as a template. TERRA is a critical component of telomere length homeostasis (6). It comprises chromosome-specific sub-telomeric sequences at the 5′ end and long tracts of UUAGGG-repeats towards the 3′ end (7–10). Owning to its G-rich sequence, TERRA forms a parallel G-quadruplex structure (11–13). Additionally, TERRA can engage in *trans* with the telomere, resulting in the formation of an R-loop (10). In this triple-strand structure, the G-rich TERRA forms an RNA:DNA hybrid with the C-rich strand and displaces the G-rich DNA strand (14). In addition, non-conventional triplex structures can form between double-stranded and single-stranded nucleotide sequences. This is exemplified by the C. *reinhardtii* telomere (TTTTAGGG)_2_, which forms a triplex structure in Na^+^ solution, stabilized with reverse Hoogsteen (G–G) and Wobble (G–T) base pairing (15). Similarly, synthetic models of *Tetrahymena* chromosomes form an intramolecular pyrimidine-purine-purine triplex, where the single-stranded G-rich overhang folds back into the major groove of the terminal duplex, forming CGG base triads in physiological buffer condition (16). Magnetic tweezers studies have revealed the formation of a stable parallel triplex between ssDNA and its freely diffused homologous dsDNA (17), as well as the formation of a stable 5% telomeric triplex structure and even a higher probability of triplex formation by GA triplex-forming oligonucleotides (5′-GGA GGA GGA GGA GGG GGA GG-3′) (18).

Among its various interactions, TERRA’s direct association with TRF1 and TRF2 plays a significant role in telomere regulation (19). TRF2, in particular, recognizes the G-quadruplex structure of TERRA through its N-terminal Gly/Arg-rich (GAR) basic domain (20). The absence of the GAR domain disrupts this interaction, culminating in abnormal TERRA localization and the formation of diffused foci at the telomeres *in vivo* (19). In contrast, TRF1, through its N-terminal acidic domain, acts to counterbalance TRF2-mediated R-loop formation (21). Cells deficient in this acidic domain exhibit R-loop accumulation and subsequent telomere instability and dysfunction (21). This delicate interplay between TERRA, the shelterin complex, and the telomeric DNA underscores the complex regulatory mechanisms essential for telomere stability and function.

Furthermore, RAD51, integral to homologous recombination and DNA repair, has also been demonstrated to promote R-loop formation at the telomeres (10). On the other hand, RNase H1 antagonizes R-loop accumulation, thereby preventing telomere fragility, particularly in ALT tumor cells in which TERRA expression is upregulated (22).

We sought to decipher the complex interplay among TERRA RNA, telomeric DNA, shelterin proteins, RAD51 and RNase H in modulating telomere structure. Employing single molecule co-localization, sm-FRET and MD simulation, we elucidate the molecular mechanism underlying TERRA’s recruitment and interactions at the telomere, providing deeper insights into telomere biology.

## Materials and methods

### Preparation of DNA and RNA oligonucleotides

HPLC purified DNA and RNA oligonucleotides with/without modification (biotin, Cy3/Cy5) were purchased from Integrated DNA Technologies (Coralville), and all the sequences are listed in Supplementary Table S1.

The oligonucleotides were resuspended in nuclease free water with stock concentration of 100 μM and stored at –20°C. The TERRA oligonucleotides (100 μM) were aliquoted in RNase free space prior to freezing to prevent degradation from freeze/thaw process.

### Protein purification

Recombinant human glutathione s-transferase-tagged POT1 was purified using baculovirus/insect cell expression system as described in (23–25). N-terminal histidine _6_-tagged TRF1, TRF2 and TRF2ΔB were purified using a baculovirus/insect cell expression system and an AKTA Pure FPLC (Cytiva) as reported previously (23,26,27). RAD51 was purified using the method described in (28). Briefly, RAD51 was expressed using the pCH1/RAD51o vector in *E. coli* Acella™ DE3 competent cells, which lack the gene encoding for RecA protein. The expressed RAD51 protein was pelleted and resuspended in lysis buffers containing protease inhibitors and lysozyme. The lysate was sonicated, and then dialyzed against spermidine acetate buffer, which precipitates RAD51. The precipitate was resuspended in increasing amounts of NaCl and the purified through a Blue Agarose Column followed by a heparin column to remove RAD51 bound DNA. Lastly, RAD51 was concentrated through a MonoQ anion exchange column.

### Sample preparation for the single-molecule FRET measurements

The telomeric duplex DNA (10μM) was prepared by mixing biotinylated C-rich strand [5′-CCCT/Cy5/AACCCTAACCCTAACCCTAAGCCTCGCTGCCGTCGCCA-3′-biotin] with its complementary non-biotinylated G-rich strand [5′-TGGCGACGGCAGCGAGGCTTAGGGTTAGGGTTAGGGTTAGGG-3′] at a molar ratio of 1:1.2 in T50 buffer (10 mM Tris–HCl, pH 7.5 and 50 mM NaCl)). The mixed oligonucleotides were annealed in a thermocycler with the following standard program as reported earlier: 95°C for 2min, then gradually cooling at the rate of 2°C/min down to 40°C followed by 5°C/min cooling to reach 4°C (23,29). The annealed stock constructs are kept at –20°C and reannealed before each experiment.

For binding assay, single-stranded oligonucleotides with an initial concentration of 100 μM were first diluted to 1 μM using T50 buffer. Proper folding of the oligonucleotides was achieved by heating them to 90°C for 2min, followed by a gradual cooling at a rate of 2°C/min until a temperature of 4°C was reached. After this thermal cycling, the samples were immediately transferred to ice and kept there throughout the experiment. This procedure was consistently applied in all experiments. Additionally, the concentration of the oligonucleotides was further verified using a NanoDrop spectrophotometer and oligo analyzer tool available on IDT website.

### Slide preparation for single-molecule assay and data acquisition

For single molecule assays, polyethylene glycol (PEG) passivated slides were prepared and assembled to microfluidic sample chamber by following the standard protocol described earlier (30). Single-molecule FRET measurements were carried out using a prism-based total internal reflection fluorescence (TIRF) microscope at room temperature (23.0 ± 1.0 °C). The Cy3 and Cy5 fluorophores were excited by a 532-nm laser (Coherent Compass 315M) and a 638-nm laser (Cobolt 06-MLD) respectively. The fluorescence emission from Cy3 and Cy5 were simultaneously collected by a water immersion objective (Olympus NA 1.2, 60×) and separated into donor and acceptor emission by a long-pass dichroic mirror (Semrock FF640-FDi01-25 × 36) followed by projected onto the electron-multiplying charge-coupled device camera (iXON, Andor Technology). Spots detection, background subtraction, donor leakage and acceptor direct-excitation was corrected as previously described (31,32). Single-molecule traces were recorded with a 100 ms time resolution and analyzed with Interactive Data Language (IDL) script. Custom codes are available on GitHub (https://github.com/Ha-SingleMoleculeLab) and archived in Zenodo with the following doi. Data acquisition DOI: 10.5281/zenodo.4925630; Raw data analysis DOI: 10.5281/zenodo.4925617.

### Single-molecule TERRA or DNA binding assay

The annealed telomeric duplex DNA was diluted to 25 pM in T50 buffer and immobilized on the PEG passivated surface via biotin-neutravidin linkage, and unbound molecules were washed out with T50 buffer. G-quadruplex (G4) substrates have a great propensity for nonspecific binding to PEG surface (33). To prevent the nonspecific binding, the PEG surface was incubated with 0.2 mg/ml BSA for 1hr at room temperature after applying neutravidin and prior to immobilizing the DNA substrate.

For binding assay, TERRA or DNA samples were diluted in T50 buffer to the desired concentration and applied to the immobilized telomeric duplex. After 10 min of incubation, unbound TERRA/DNA were washed out three times using T50 buffer. Single - molecule measurements were then performed in an imaging buffer containing 10 mM Tris–HCl, pH 7.5, 100 mM NaCl, 10% glycerol with an oxygen scavenging system (10 mM Trolox, 0.5% glucose, 1 mg/ml glucose oxidase and 4 μg/ml catalase).

For experiments involving shelterin proteins, the proteins were incubated for 15 min to allow sufficient binding and the unbound proteins were washed with T50 buffer prior to the addition of TERRA or TERRA-18. To ensure direct comparison in the binding assay with RAD51, both G4-18 or TERRA-18 samples and the G4-18 or TERRA-18 with RAD51 samples were treated identically. Each sample was first incubated at 37 °C for 10 min in binding buffer comprising 50 mM Tris–HCl (pH 7.5), 1 mM CaCl_2,_1 mM MgCl_2_, 1 mM AMP-PNP. This step was followed by 10 minutes incubation with a tethered telomeric duplex in the reaction chamber. After the incubation, each sample was washed three times with T50 buffer and measurement was performed in an imaging buffer.

**Digestion assay by RNase H, DNase I** (New England BioLabs) was used at a concentration of (20units/mL) and incubated for 10min to treat the triplex structure. Proteinase K (New England BioLabs) was applied at a concentration of 16units/mL and incubated for 10 minutes to digest TRF2 and RAD51 proteins. Following digestion with RNase H, DNase I and proteinase K, samples were washed with T50 buffer and measurement was performed in imaging buffer.

### Single-molecule assay for TERRA transcription

The labeled TERRA transcription top and bottom strand were annealed with biotinylated 18-mer DNA (Supplementary Table S1) in 10 mM Tris–HCl (pH 8.0) and 5 mM MgCl_2_ containing buffer at the ratio of 1:1.2:1.5, respectively. Single-molecule TERRA transcription was performed in an imaging buffer containing 40 mM Tris–HCl, pH 8, 50 mM KCl, 6 mM MgCl_2_, 1 mM DTT, 2 mM spermidine and 0.1 mg/ml BSA with an oxygen scavenging system. TERRA transcription was initiated by applying T7 RNAP (1.25 units/μl) mixed with rNTP (1 mM) to the immobilized DNA. Real-time R-loop and G-quadruplex formation during transcription was measured by collecting long (∼180 s) and short (∼2 s) movies with different time intervals. For RNase treatment, the imaging buffer was mixed with RNase H (final concentration 0.05 U/μl).

### Single-molecule data analysis

The trace outputs were processed with custom MATLAB script to generate single-molecule time trajectories and FRET histograms. Each FRET histogram was generated by collecting FRET values from at least 4000 molecules taken ∼20 short movies collected from different imaging areas. Donor-only contribution was corrected from the histogram at the low FRET region and the histograms are fitted with Gaussian distribution function using Origin software. For single molecule colocalization assay, 21 movies (40 frames each) were recorded from different imaging surfaces. For each movie the sample was excited with Cy3 laser (20frames) followed by Cy5 laser (20 frames). A python script was used to quantify the Cy3–Cy5 colocalization using the equation-1 to measure Cy3 labeled TERRA/DNA binding to the immobilized Cy5 labeled telomeric duplex DNA.


(1) \begin{eqnarray*}\frac{{Cy3.Cy5{\mathrm{\ }}colocalization{\mathrm{\ }}\left( {FRET} \right)}}{{Cy5}}\end{eqnarray*}

### Electrophoretic mobility shift assay (EMSA)

EMSA for RAD51 binding to G4-18, TERRA-18, G4-DNA(8oxoG)-18, ssRNA (poly 40 nucleotides uracil) was performed by adapting the protocol (10). The stock of RAD51 protein was diluted in a buffer containing 25 mM Tris–HCl (pH 7.5), 10% (v/v) glycerol, 0.5 mM EDTA, 50 mM KCl, 1 mM DTT and 0.01% NP40 incubated with the DNA or RNA substrates at 37 °C for 10 min in a buffer that contains 50 mM Tris–HCl (pH 7.5), 50 mM KCl, 1 mM MgCl_2_, 1 mM ATP (10). The products were resolved using 0.8% TBE agarose gels supplemented with 10 mM KCl at 4 °C for 50 min (6.5 V cm^−1^). Gels were imaged on Amersham imager 600 and analyzed with ImageJ.

### Spectrofluorometer

The emission spectra of Cy3 and Cy5 (FRET) with Cy3 excitation before and after adding an excess of unlabeled TERRA-18 (1 and 2 μM) to the Cy3–Cy5 labeled DNA duplex (100 nM) was measured by using cary eclipse fluorescence spectrophotometer for 10 min.

### Modeling and simulation method

To probe the interactions between the DNA duplex and TERRA strand, we performed coarse-grained simulations followed by all-atom molecular dynamics (MD). SimRNA (34), a coarse-grained method based on replica exchange Monte Carlo simulation (REMC), was used to generate an initial structure ensemble of the complex. A total number of 40 replicas were used for REMC, with each replica lasting 1e^7^ steps and the temperature spanning from 1.5 to 1.0. Weak DNA–DNA and DNA–RNA pairing potentials were applied to facilitate the sampling. The lowest energy conformations (6% of the total frames) were used for clustering analysis, after aligning the complex to the TERRA/C-rich region. The centroid structures of top 10 representative clusters were chosen for the subsequent all-atom MD simulations. Each DNA-RNA system was solvated in a water box with 150 mM NaCl. All the simulations were performed using the GPU version of Amber18 (35) with the OL15 parameters for DNA (36) and OL3 parameters for RNA (37). Firstly, energy minimization was performed to eliminate steric clashes. A following 10 ns equilibration MD was performed under isothermal-isobaric conditions at 1 bar and 300K, with harmonic restraints applied to nucleic-acid atoms using a spring constant of 0.1 kcal/(mol Å^2^). The temperature was maintained by Langevin dynamics with a friction coefficient of 1 ps^−1^. Next, Gaussian accelerated MD (GaMD) (38) was employed to enhance the sampling of nucleic-acid conformational dynamics as follows. A 10-ns conventional MD stage was first used to gather statistics for determining the initial GaMD acceleration parameters. Then a 40-ns GaMD equilibration stage was conducted, followed by a production GaMD run which lasted 300 ns. Both total potential energy boost and dihedral energy boost were applied to the system, each having a 6 kcal/mol upper limit of the standard deviation to ensure accurate reweighting.

## Result

### Sub-telomeric sequence promotes TERRA association with telomeric DNA

**Figure 1.**
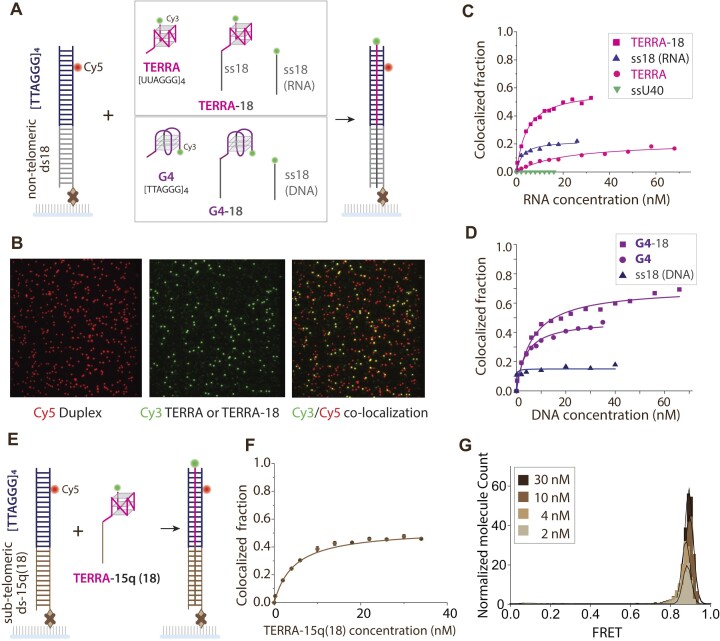
TERRA association with telomeric duplex DNA is enhanced by sub-telomeric sequence. DNA construct: (**A**) Cy5 labeled telomeric duplex [TTAGGG]_4_ with 18mer bp located adjacent to telomeric duplex is immobilized. Cy3-TERRA, Cy3-TERRA-18 or Cy3-ss18(RNA) is applied to the telomeric DNA. TERRA RNA consists of [UUAGGG]_4_, and 18 is a preceding RNA sequence complementary to the 18bp located adjacent to the telomeric duplex. The Cy3-G4, Cy3-G4-18 and ss18 DNA are the DNA counterparts to Cy3-TERRA, Cy3-TERRA-18 and ss18 RNA, respectively applied to the immobilized DNA. (**B**) Colocalization between the Cy3 and Cy5 serves as a readout to quantify binding of TERRA to the telomeric duplex. (**C**) Comparison of the annealing of TERRA-18, TERRA, ss18(RNA) or ssU40 to a telomeric duplex with 18mer base. (**D**) Comparison of the annealing of TERRA-18, TERRA and ssRNA to the DNA counterpart G4-18-DNA, G4-DNA and ssDNA, respectively. (**E**) DNA construct: Cy5 telomeric duplex [TTAGGG]_4_ with 15q (18) bp located adjacent to telomeric duplex. Cy3-TERRA- 15q (18) consisting of [UUAGGG]_4_ and a precedint 15q(18) sequence complementary to the 15q 18bp located adjacent to telomeric duplex is applied to the immobilized DNA. (**F**) The fraction of colocalization of TERRA-15q and telomeric duplex with 15q (18) base. (**G**) Histogram for the binding TERRA-15q (18) at 2, 4,10 and 30 nM concentrations to telomeric duplex with 15q (18) basepairs.

The TERRA sequence, 5′-UUAGGG-3′ repeats, is necessary and sufficient to drive its association with telomeres in *trans* (10). The telomere consists of a duplexed [TTAGGG]_4_ region and a 3′ single-stranded DNA overhang, which contains TTAGGG tandem repeats. This overhang folds into a compact G-quadruplex (G4) structure (23,39–42). Here, we employed a single molecule colocalization assay to measure the physical association of TERRA and telomeric DNA. We prepared a DNA construct that contains a telomeric duplex [TTAGGG]_4_ labeled with a Cy5 dye for visualization and a biotin for immobilizing to a PEG-passivated surface. We then applied Cy3 labeled TERRA or telomeric overhang (G4) to the immobilized DNA. TERRA is a single-stranded (ss) RNA consisting of [UUAGGG]_4_; the G4 is the telomere overhang sequence bearing [TTAGGG]_4_. We first tested annealing of TERRA or G4 without and with a sub-telomeric sequence by adding an 18-mer extension to both TERRA and G4 that bears a complementary sequence to the non-telomeric 18bp region of the immobilized DNA. In addition, we applied an 18-mer RNA or DNA alone as a control (Figure 1A). We illuminated the single molecule surface with both the green (532 nm) and red (633nm) lasers to localize both Cy3 and Cy5 signals independently and quantified the ratio of colocalization ($\frac{{cy3.cy5}}{{cy5}}$) to measure the binding efficiency of TERRA to the telomeric duplex (Figure 1B).

TERRA and G4 bound to telomeric duplex with 15% and 40% binding efficiency, respectively (Figure 1C, D). The TERRA and G4 association was sequence-specific as the poly-uracil (U40) 40-mer produced no Cy3 colocalization with Cy5 (Figure 1C). Interestingly, G4 exhibited higher binding efficiency than TERRA. This could be due to the higher stability exhibited by RNA quadruplexes compared to DNA quadruplexes, attributed to noncovalent interactions and the presence of extra 2′-OH group in RNA (43–45). For both TERRA and G4, the addition of the complementary 18-mer (TERRA-18, G4-18) significantly increased the binding efficiency from ∼15% to ∼ 50% for TERRA and 40% to 65% for G4 (Figure 1C, D, Supplementary Table S2). Since the 18-mer was not derived from a human sub-telomeric sequence, we also tested the effect of the last 18 nucleotides in the sub-telomeric region of chromosome 15q (Supplementary Figure S1A) used in a previous study (10). The binding efficiency of TERRA-15q (18) to telomeric duplex with 15q (18) base is ∼50%, on par with the TERRA-18 (Figure 1E, F and Supplementary Figure S1B, C), signifying the role of an extended complementary strand, but not the specific sequence of the sub-telomere in promoting TERRA association. Due to the proximity between the Cy3 and Cy5 that results from TERRA-telomere interaction, we can also monitor the binding by plotting a FRET histogram as a function of TERRA-15q (18) concentration (Figure 1G).

Replacing the 18mer with a poly U (TERRA-18poly U) slightly increased TERRA binding efficiency by ∼ 10%, underscoring sequence specific nature of this effect (Supplementary Figure S1D, E). Next, we tested if RNA or DNA, lacking TERRA or G4 sequences, associates with the non-telomeric 18 bp region. While nonspecific ssU40 showed no binding, the sequence-matched ss18(RNA) and ss18(DNA) each showed ∼20% binding, reflecting its role in enhancing the affinity of TERRA and G4 (Figure 1C, D). Taken together, the results suggest that the extended sequence moderately enhances binding by potentially destabilizing the compact G-quadruplex structure and also open up the tightly base-paired DNA duplex and thereby facilitate the invasion of TERRA. Next, we asked if TERRA is stretched out when bound to the telomere as it is expected to fold into G-quadruplex on its own (11–13). To test the TERRA conformation, we labeled both ends of TERRA with Cy5 and Cy3 and applied to unlabeled telomeric duplex DNA. The resulting FRET histogram shows a single low FRET peak, indicating that TERRA unfolds when *trans*-annealed to the telomeric duplex (Supplementary Figures S1F–H).

### TERRA exhibits dynamic mobility on telomeric duplex, which is stabilized by TRF2

**Figure 2.**
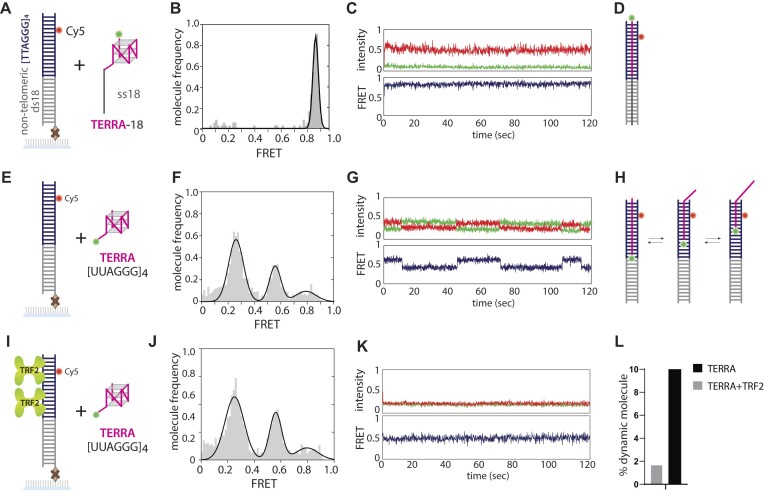
TERRA without sub-telomeric sequence exhibits dynamic states that are stabilized by TRF2. (**A–D**) smFRET assay shows high (0.9) stable FRET when Cy3-TERRA-18 is applied to Cy5-telomeric DNA. (**E, F, H**) Cy3-TERRA application to the DNA yields three FRET histogram peaks. (**G, L**) The individual smFRET traces undergo FRET transition and dynamics. (**I, J**) FRET peak distribution In the presence of TRF2. (**K, L**) The individual smFRET traces with the addition of TRF2.

We used single-molecule colocalization and smFRET to demonstrate that TERRA and G4 *trans* anneals to the telomeric duplex, as shown above. Next, we examined the FRET histograms and single molecule traces to monitor the bound state of TERRA-18 versus TERRA. TERRA-18 binding yielded a single high FRET peak, indicating a single conformation of the RNA–DNA complex (Figure 2A, B). Consistently, most single-molecule time traces exhibited a stable high FRET state (Figure 2C). Both results reflect that TERRA-18 molecules *trans*-anneal to the entire length of the telomeric duplex with the 18bp stem (Figure 2D).

In contrast, TERRA without the extended single-strand RNA (18) produced three peaks, which likely represent the differently annealed state of TERRA to telomere (24, 18, 12 nucleotides) due to the nature of the tandem repeat sequences (Figure 2E, F, H). Unexpectedly, approximately 10% of smFRET traces exhibited dynamic conversions between different FRET states. This FRET fluctuation persisted after completely removing excess TERRA, ruling out the possibility of rebinding (Figure 2G, L). In addition, the motion did not depend on the salt concentration, which is consistent with a 1D sliding movement (Supplementary Figure S2A, B). When the same assay was performed in the presence of TRF2, which is associated with the telomeric DNA (2,23,46,47), a similar FRET peak distribution was obtained (Figure 2I, J). Unlike the case without TRF2, however, the smFRET traces displayed mostly static FRET states with only about ∼2% showing dynamics (Figure 2K, L). This suggests that TRF2 stabilizes the annealed state of TERRA.

### TERRA *trans* annealing results in R-loop formation

**Figure 3.**
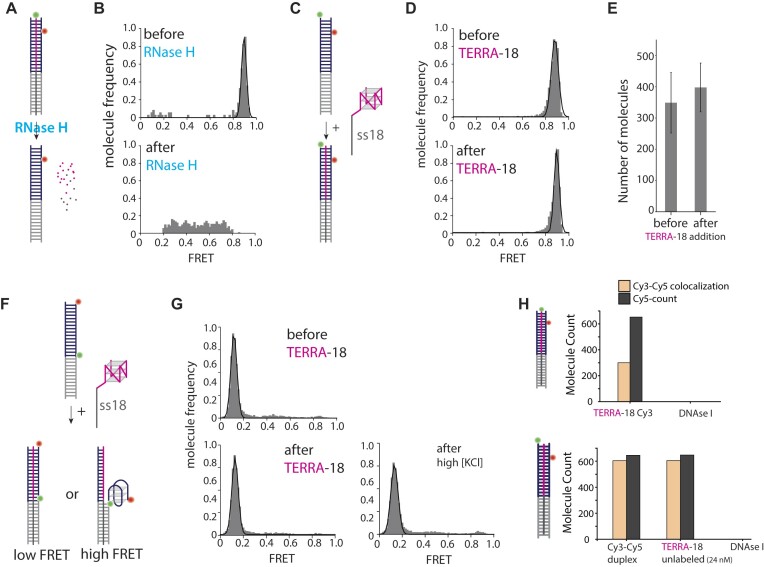
TERRA annealing results in RNA-DNA hybrid formation without displacing the G-rich strand. (**A, B**) Cy3-TERRA-18 is applied to Cy5 telomeric DNA. SmFRET histograms show loss of high FRET after TERRA is digested by RNase H. (**C, D**) Unlabeled TERRA-18 (1 uM) applied to Cy3–Cy5 labeled telomeric DNA. Sm (FRET) histogram and traces show stable high FRET before and after unlabeled TERRA-18 is applied. (**E**) The molecule count of the Cy3-Cy5 duplex remains the same before and after the application of excess unlabeled 18-TERRA. (**F**) The G-rich strand is labeled with Cy3 and Cy5 (Cy5- [TTAGGG]_4_ -Cy3), and excess unlabeled TERRA is applied. Schematic shows prediction that low FRET remains if G-quadruplex does not form in DNA, and transition to a high FRET if G-quadruplex structure forms. (**G**) Upon applying excess unlabeled TERRA-18 and incubating with 100 or 150 mM KCl the FRET remains in the low state. (**H**) The same measurement as in panel C performed in ensemble fluorescence. Green and red lines represent fluorescence signals obtained from Cy3 and Cy5 over time.

So far, we monitored the association of TERRA to telomeric DNA by both colocalization and smFRET. Here, we asked if the physical interaction between TERRA and telomeric duplex results in an R-loop formation. An R-loop is a triple-stranded structure consisting of an RNA-DNA hybrid and a displaced single-stranded DNA (14).Therefore, TERRA is expected to hybridize with the C-rich strand while the G-rich strand is displaced. As before, Cy3-labeled TERRA applied to Cy5-labeled telomeric duplex results in a single high FRET peak (Figure 3A, B, top). The high FRET peak completely disappeared immediately after we added RNase H, which selectively digests the RNA from RNA: DNA hybrids (Figure 3B, bottom), signifying that TERRA formed a hybrid with the C-rich DNA strand.

Next, we tested if the G-rich strand is displaced due to the RNA: DNA hybrid formation. We placed Cy3 and Cy5 on G-rich and C-rich strands, respectively, and applied a molar excess of TERRA-18, which exhibited the highest efficiency of engagement with the telomere DNA shown in Figure 1C. The high FRET state observed from the duplex DNA-only condition was maintained even after the addition of unlabeled TERRA-18 up to 1 μM concentration (Figure 3C, D), and the number of G-rich strands (non-biotinylated) on a single molecule surface remained the same before and after the unlabeled TERRA-18 addition (Figure 3E). Both results indicate that the G-rich strand is not displaced from the RNA:DNA hybrid structure. In a parallel ensemble experiment using a spectrofluorometer, we monitored the emission of Cy3 and Cy5 (FRET) with Cy3 excitation before and after adding an excess of unlabeled TERRA-18 (1 and 2 μM) to the Cy3-Cy5 labeled duplex (Supplementary Figure S3A). Consistently, no significant change was noted in the emission of Cy3 and Cy5 (FRET) (Supplementary Figure S3B). Had the G-rich strand been displaced, a notable decrease in FRET efficiency would have been expected due to the altered proximity between Cy3 and Cy5. These results collectively suggest that the G-rich strand remains intact during TERRA-18 engagement with the telomeric duplex comprising an 18 bp. We further tested if the hybrid formation would partially release the G-rich strand to fold into a G-quadruplex. To test this, FRET dyes were attached across the G4 forming sequence such that G4 folding would produce high FRET (Figure 3F). The low FRET persisted even after the addition of unlabeled TERRA-18 and also in the presence of KCl, which promotes G4 formation (48–50) (Figure 3G). This observation remained consistent even when TERRA-18 was applied together with POT1, a shelterin protein known to bind to the G4 structure (23,25,51) (Supplementary Figure S3C). Taken together, the G-rich strand remains intact even when TERRA anneals to the C-rich strand and reveals that TERRA is triplex-forming oligonucleotide (TFO).

We then examined the susceptibility of the telomere-triplex structure to DNase I mediated hydrolysis. DNA engaged in RNA: DNA triplex structure is expected to show resistance to hydrolysis by DNase I (52). With the Cy5 dye on the C-rich strand and the Cy3 dye either on the TERRA-18 or the G-rich strand, the structure was treated with DNase I (Figure 3H). As expected, when the Cy3 dye is on TERRA-18 the ratio of ($\frac{{cy3.cy5}}{{cy5}}$) co-localization is ∼50% and when unlabeled TERRA-18 is applied to Cy3–Cy5 labeled telomere duplex the Cy3–Cy5 co-localization count remains unchanged. Interestingly, upon digestion with DNase I and normalizing to a digestion of DNA duplex by DNase I, a complete loss of Cy3–Cy5 co-localization count and Cy5 count was observed in both experiment setups. This suggests that despite the presence of the triplex structure, there remains DNA duplex accessible for DNase I digestion. These results imply formation of the triplex structure in the telomere region, leaving the 18bp region of the DNA duplex vulnerable to DNase I digestion.

**Figure 4.**
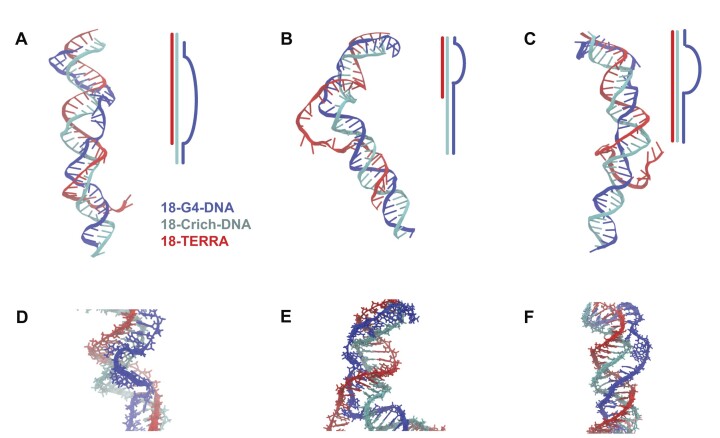
Structural variations of the DNA-TERRA complex revealed by combining coarse-grained and all-atom simulations. (**A–C**) Three representative structures of the system based on clustering the molecular simulation results. Strand-strand interactions are demonstrated by a schematic, highlighting R-loop and triplex formation. (**D–F**) A close-up view of the R-loop region.

To characterize the atomistic-level picture of the dsDNA-TERRA complex, we performed coarse-grained modeling followed by all-atom MD simulations (see Materials and methods). The revealed conformational ensemble is diverse, and we were able to observe R-loop and triplex formation. Figure 4 demonstrates three representative structures from clustering analysis. In each structure, an R-loop region was observed where the TERRA strand (red) forms a hybrid duplex with the Crich-DNA by displacing the G4-DNA (blue). The locations and lengths of the R-loop region vary among the structures (Figure 4A-C). Atomistic view of each R-loop region is shown in (Figure 4D–F). R-loop generally forms at the upper region of the 18-G4-DNA. TERRA insertion occurs in the middle or lower region of the strand, while the lower part remains as a DNA duplex.

### TERRA transcription induces R-loop and G-quadruplex formation

**Figure 5.**
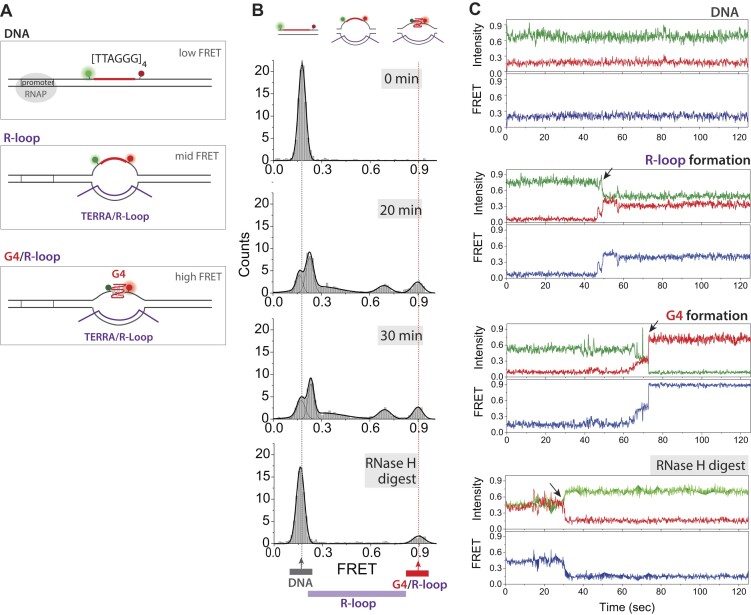
Structural variations of the DNA-TERRA complex revealed by combining coarse-grained and all-atom simulations. (**A**) FRET construct to monitor R- loop (mid-FRET) and G4 (high FRET) formation. (**B**) FRET level before transcription, in 20 minutes and 30 minutes of transcription and after RNase H treatment. (**C**) Real-time single molecule traces for DNA-only, low to mid FRET transition for R-loop formation, low to mid to high stepwise FRET transition from DNA to R-loop to G4 and mid to low FRET transition upon RNase H digestion of the R-loop.

A previous study has demonstrated that telomeric R-loops increase the formation of G-4s at the telomeres (53). To test another biological context, we performed an experiment in which TERRA is transcribed directly from telomeric DNA template. We have a well-established single molecule assay designed for probing R-loop and G4 presented in our previous work (54). For TERRA transcription measurement, we prepared DNA substrate which contains a promoter for T7 RNAP and a TERRA coding sequence, [TTAGGG]_4_ (Figure A, top). We positioned Cy3 and Cy5 dyes across the [TTAGGG]_4_ on the non-template strand for FRET measurement. This FRET construct enables us to monitor the R-loop (mid FRET) and G4 (high FRET) formation during transcription (Figure 5A).

The FRET level before transcription is ∼0.25 due to the 24 bp separation between the two dyes (Figure 5B, 0 min). We initiate transcription by applying T7 RNAP (1μM) and NTP (1mM). In 20 minutes of transcription, we observe a small mid FRET peaks (∼0.3–0.7) and a distinct high FRET peak (∼0.9), corresponding to R-loop and G4, respectively (Figure B, 20 min). The pattern becomes more distinct over 30 minutes of transcription reaction. When we add RNase H which selectively degrades RNA within R-loop, the mid FRET peaks completely disappear while the high FRET peak remains, confirming the mid- and high FRET peaks as R-loop and G4 structures.

Real-time single molecule traces consistently show the steady low FRET for DNA-only (Figure 5C, top), low to mid FRET transition for R-loop formation (Figure C, second), low to mid to high FRET representing a stepwise transition from DNA to R-loop to G4 state (Figure C, third) and mid to low FRET transition due to RNase H digestion of the R-loop (Figure C, bottom). Together, this result signifies that the R-loop that forms as a result of TERRA transcription can induce G4 on the non-template DNA, emphasizing that if the R-loop induces G4 is context dependent.

### G4 overhang enhances TERRA annealing

**Figure 6.**
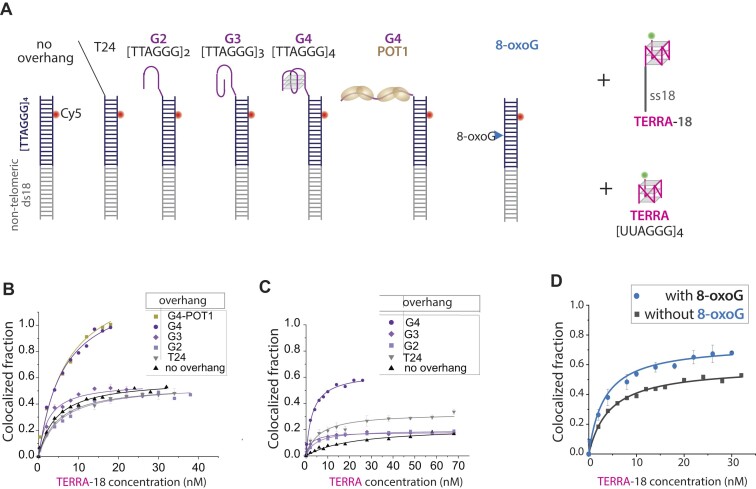
Telomeric DNA overhang and an 8oxoG lesion in the telomere duplex enhances TERRA binding. (**A**) Cy3 labeled TERRA or TERRA-18 was applied to telomeric duplex with or without various overhangs (T24, G2, G3, G4) constructs that are Cy5 labeled. Cy3-TERRA-18 was applied to the telomeric duplex with G4 overhang prebound to POT1 and telomeric duplex with an 8oxoG damage and Cy5 labeled. (**B, C**) Fraction of colocalization between TERRA-18 or TERRA with the different DNA constructs and G4 overhang prebound to POT1 as indicated. (**D**) Fraction of colocalization of TERRA-18 with telomeric duplex with or without 8oxoG damage.

Building on the understanding of the telomere 3′ single-stranded DNA overhang and its capacity to form a G-quadruplex (G4) structure, we asked if TERRA *trans* annealing to telomere duplex is affected by the presence of the G4 telomeric overhang on the duplex and if TERRA interacts with the G4 overhang. To test this, we prepared a telomeric duplex with variable overhang composition (no overhang, T24, G2, G3, G4) (Figure 6A). The G4 overhang [TTAGGG]_4_ folds into a G-quadruplex structure, while the T24 (poly-thymine, 24nt) is the same length with no structure. G2 and G3 constructs have two and three repeats of TTAGGG overhang, which are used to test overhang length dependence for TERRA binding. We observed that the colocalization significantly increased for both TERRA-18 and TERRA by the duplex having a G4 overhang, while other overhang constructs had a negligible effect on TERRA association (Figure 6B, C). Strikingly, 100% colocalization is achieved in the presence of both the G4 overhang on telomere DNA and the 18 sub-telomeric sequence on TERRA, signifying an optimal condition that favors *trans* annealing.

We asked if TERRA annealing is enhanced due to a direct interaction with the G4 overhang on the duplex. To test TERRA and G4 interaction, we added Cy3-labeled TERRA to Cy5-labeled G4 overhang on a non-telomeric duplex. The lack of colocalization up to 12 nM TERRA revealed no direct interaction between TERRA and G4 overhang (Supplementary Figure S4A). This confirms that the increased annealing is not due to the direct interaction between TERRA and G4 overhang. Based on these results, we hypothesized that the G4 overhang may play a role in fraying the duplex at the junction of duplex and single-stranded DNA. T24, G2 or G3 constructs may be less effective in fraying the duplex junction since the strands are unstructured and thus spread out.

We tested for a possible fraying effect by applying POT1 to the G4 overhang. POT1 binds telomere overhangs, which can add to the bulkiness of the overhang structure (23,25,51). TERRA-18 was applied to a telomeric duplex with POT1 bound to the G4 overhang (Figure 6B, Supplementary Figure S4B). POT1 bound overhang elevated TERRA-18 annealing to a level comparable to the G4 overhang. Therefore, we hypothesize that the G4 overhang and the overhang bound to POT1 exhibit a molecular fraying effect that opens up the duplex, making it more accessible for TERRA binding.

We next tested if the telomere duplex integrity affects TERRA annealing by introducing a single 8-oxoguanine (8oxoG). 8oxoG is a common oxidative base lesion that disrupts the duplex structure due to changes in the free energy of an 8oxoG:C base pair with decreased enthalpy as the modification results in changes in hydrophilicity of the base and cation binding to major groove (55). Furthermore, telomeres are hypersensitive to 8-oxoguanine formation due to the G-rich sequence (56). The presence of the 8oxoG increased the binding of TERRA-18 by about 20% (Figure 6D), indicating that destabilization of the telomeric duplex by the 8oxoG lesion facilitates TERRA annealing.

### TRF2 promotes TERRA binding and protects TERRA from RNase H digestion

**Figure 7.**
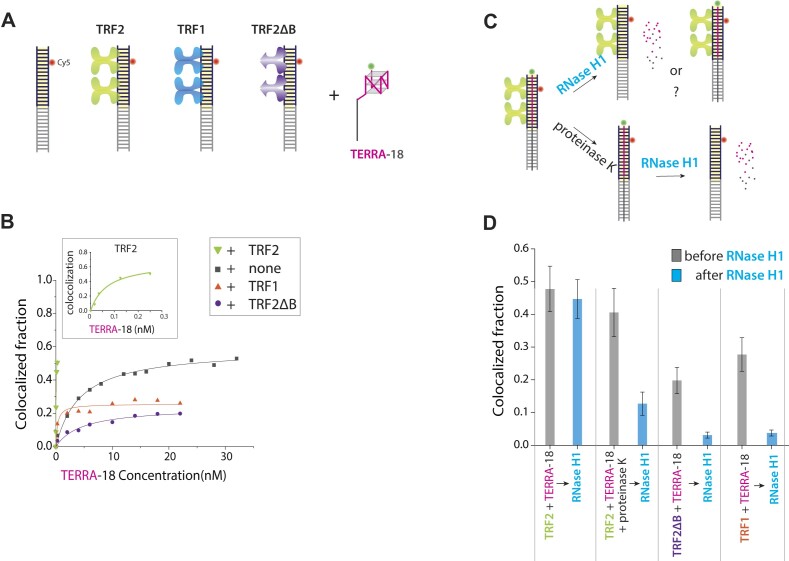
TRF2 promotes TERRA binding and protects TERRA R-loop from RNase H digestion. (**A**) Schematic of Cy3 labeled TERRA-18 applied to telomeric duplex prebound to TRF2, TRF1, or TRF2ΔB. (**B**) Colocalized fraction collected in varying protein conditions. (**C**) Experimental procedure of RNase H treatment alone (top) vs. proteinase K, followed by RNase H treatment (bottom). Proteinase K treatment is used to degrade the bound proteins. (**D**) The colocalization fraction was collected before and after the RNase H or proteinase K + RNase H treatment in all conditions. The error bar shows a standard deviation of the mean (*n* = 21).

We asked if TRF1 and TRF2, two shelterin proteins that bind telomeric duplex, alter TERRA engagement and if the proteins protect the annealed TERRA from RNase H digestion. As before, Cy3-TERRA-18 was applied to immobilized Cy5 telomeric duplex prebound with TRF2, TRF1 or a TRF2 mutant that lacks the basic domain (TRF2ΔB) proteins (Figure 7A). TRF2 significantly enhanced the binding affinity of TERRA and TERRA-18 to the telomeric duplex, achieving ∼50% binding efficiency at as low as 0.25 nM TERRA-18. Furthermore, the interaction between TERRA and TRF2 does not lead to the unfolding of the TERRA G4 structure (Supplementary Figure S5B, C). This suggests that TRF2’s role in increasing TERRA binding efficiency is likely through enhanced recruitment of TERRA, rather than by inducing structural changes in the TERRA G4 complex (Figure 7B and Supplementary Figure S5A). Conversely, TRF1 and TRF2ΔB significantly suppressed TERRA binding (Figure 7B). These results are consistent with a previous study, which showed that TRF2 interacts with TERRA through its basic domain, enhancing R-loop formation, and TRF1 prevents TRF2 interaction with TERRA and R-loop formation through its acidic domain (21). In addition, our results suggest that TRF2ΔB repels TERRA binding, lowering the annealing below that of a naked telomeric duplex. This finding is consistent with a cellular study that demonstrated the ectopic expression of TRF2 increases TERRA-Telomere colocalization while the ectopic expression of TRF2ΔB induced aberrant localization of TERRA that is diffused and formed fewer foci at the telomeres compared to the TRF2 expressing cells (19).

Next, we asked if TRF2, TRF1 or TRF2ΔB proteins protect TERRA R-loop by treating with RNase H in the presence of the proteins. The colocalization before and after the RNase H treatment showed that the TRF2 condition retained most of the colocalized TERRA, revealing the role of TRF2 in protecting the annealed TERRA. The protection was lost after the proteinase K treatment, which degrades TRF2, confirming the role of TRF2 in preventing R-loop degradation (Figure 7C, D, Supplementary Figure S5A). By contrast, TRF1 and TRF2ΔB proteins did not protect TERRA from RNase H treatment (Figure 7D). Therefore, the TRF2 basic domain is essential both for increasing the affinity of TERRA to telomere and protecting TERRA against digestion by RNase H.

### RAD51 binds TERRA with high affinity without unfolding the G4 structure and promotes its annealing to the telomere

**Figure 8.**
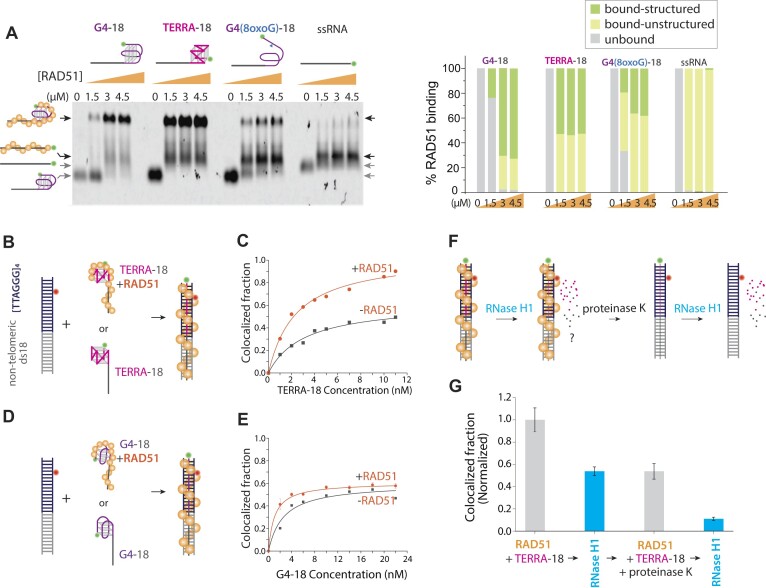
RAD51 binds TERRA with high affinity without unfolding the G-quadruplex structure and promotes its annealing to the telomere. (**A**) EMSA gel showing RAD51 binding to TERRA-18, G4-18 DNA, G4 -18–8oxoG, poly 40 oligos. (**B, C**) SM colocalization comparing annealing of TERRA-18 to the telomeric duplex with and without precomplexing with RAD51 protein. (**D, E**) SM colocalization comparing annealing of G4-18 DNA to the telomeric duplex with and without precomplexing with RAD51 protein. (**F, G**) TERRA pre-complexed to RAD51 colocalization with telomere DNA and RNase H treatment pre and post-proteinase K digestion of RAD51. The error bar shows a standard deviation of the mean (*n* = 21).

Next, we measured the impact of RAD51 in TERRA *trans* annealing. Unlike TRF2, a resident protein in telomeric DNA as a member of shelterin complex, RAD51 is a trans-acting factor that can recruit TERRA to telomeres. First, we performed EMSA to test the binding of RAD51 to G4-18, TERRA-18 and G4-18 with an 8oxoG lesion and poly-uracil 40 (similar length to TERRA-18). The two lower bands correspond to folded (lower) and unfolded (higher) RNA/DNA substrates unbound by protein (Figure 8A, gray arrows). In comparison, the two higher bands represent RAD51 bound unfolded (lower) and folded (higher) substrates (Figure 8A, black arrows). Consistent with a previous report (10), TERRA-18 has a greater affinity for RAD51 than its DNA counterpart G4-18. The highest affinity is evident from the disappearance of the unbound band at the lowest RAD51 concentration (1.5 μM) for TERRA-18 but not in other substrates. For the RAD51 bound bands, we interpreted the lower band as RAD51 bound to a stretched-out RNA (same position as the poly U40) and the upper band as RAD51 bound to G4 folded RNA and DNA. Based on the band intensity, RAD51 associates with the folded TERRA and G4 more than the unfolded strands. Interestingly, since both lack structure, RAD51 binding to G4 containing an 8oxoG and unstructured U40 primarily produced the lower bands.

The single-molecule colocalization assay revealed that the precomplexing of TERRA-18 with RAD51 greatly enhanced its annealing to the telomeric duplex (Figure 8B, C), consistent with a previous study (10). However, precomplexing G4-18 DNA with RAD51 did not produce a significant difference (Figure 8D, E). This may be related to the higher affinity of RAD51 for TERRA-18 than for G4-18 DNA. The RNase H digestion result reflects that RAD51 partially protects TERRA against the RNase H degradation, which disappears when the proteinase k treatment removes RAD51 (Figure 8F, G). Overall, our results demonstrate that RAD51 associates mostly with G4 folded TERRA structures, RAD51-TERRA complex exhibits enhanced annealing efficiency to telomeric duplex, and RAD51 can partially protect TERRA from the RNase H digestion.

## Discussion

The intricate interplay between TERRA, telomeric DNA, shelterin proteins, and other factors involved in telomere maintenance is fundamental to ensuring telomere integrity. Our findings provide molecular insights into the physical interaction between TERRA and telomeric DNA and highlight the modulatory roles the shelterin proteins, RAD51 and RNase H play in the process. Our data reveals that the association between TERRA and the telomeric DNA is highly sequence-specific and significantly enhanced by a sub-telomeric sequence. This finding demonstrates that the additional sequence beyond the repetitive telomeric region can influence TERRA’s ability to anneal and interact with the telomere, potentially impacting its role in telomere homeostasis. Cellular TERRA is expected to contain the sub-telomeric sequence since the TERRA transcription starts from a sub-telomeric region (7). Using an 18-mer extension to both TERRA and a (TTAGGG)_4_ ssDNA overhang resulted in ∼15% to ∼50% and 40% to 65% increase in binding efficiency respectively, indicating that this complementary sequence could facilitate the unwinding or destabilization of the DNA duplex, allowing for more efficient strand invasion. We observed similar binding efficiency using a 15q sub-telomeric sequence from chromosome 15q, reinforcing that the extended complementary region rather than a specific sub-telomeric sequence is crucial for enhancing association. Our results suggest that TERRA unfolds from its G-quadruplex structure when *trans-*annealed to the telomeric duplex. Additionally, the presence of an RNA sequence preceding the TERRA sequence increases the unfolding of TERRA as a non-specific polyU increased annealing, albeit to a lesser degree than a complementary sequence.

Interestingly, our single-molecule assays reveal that the *trans* association of TERRA with the telomeric duplex does not displace the G-rich strand but, instead, forms a stable triplex structure. This finding is also supported by all-atom MD simulations. Additionally, TERRA transcription from telomeric DNA induces R-loops, which promote G-quadruplex (G4) formation, highlighting the context-dependent nature of triplex structure formation. Triplex structures play significant roles in various biological processes, including transcription coupled DNA repair (57), epigenetic modifications (58), replication fork stalling (59), homologous recombination (17), and telomere stability (16). At the telomere, such a triplex structure could potentially serve as a protective mechanism against endonuclease digestion (16) of the displaced strand and subsequent activation of the DNA damage repair pathway. The triplex structure could facilitate the recruitment of specific proteins or serve as an intermediate in homologous recombination processes (17). Further studies are required to dissect the molecular details of this triplex structure, elucidate its functional roles, and understand its implications for telomere maintenance.

The G-quadruplex structure at the 3′ single-stranded DNA overhang of telomere plays critical roles in telomere protection and regulation of telomerase activity (39,60,61). In our study, introducing a telomeric G4 overhang led to a striking increase in TERRA annealing, underscoring the potential significance of the G4 structure in modulating TERRA interactions at the telomeres. Interestingly, our data suggests that the enhanced TERRA annealing in the presence of a G4 overhang does not result from direct interaction between TERRA and G4 structure. This is a critical distinction, as it points towards a more indirect mechanism of action, potentially involving the fraying of the telomeric duplex at the junction between the double-stranded and single-stranded regions. This fraying effect, which persists when POT1 occupies the overhang, could result in increased accessibility of the telomeric DNA to TERRA, thereby promoting annealing. Furthermore, the enhanced annealing efficiency in the presence of 8-oxo guanine (8oxoG), a frequent oxidative base lesion, is consistent with the idea that destabilization of the telomeric duplex by 8oxoG promotes TERRA annealing, reinforcing the notion that the structural integrity of the telomeric DNA is a key determinant of TERRA interaction.

The shelterin complex, with its various components, including TRF1 and TRF2, is integral to telomere maintenance, protecting the ends of chromosomes and regulating telomerase activity. TRF2, in particular, is essential for preventing inappropriate DNA damage responses at telomeric ends (62–64). Building upon this knowledge, we provide evidence that TRF2 not only enhances TERRA’s affinity for the telomeric duplex to form a triplex but also protects the annealed TERRA in the triplex from RNase H digestion. This dual role of TRF2 underscores its importance in maintaining a stable and protective telomeric structure. Conversely, we demonstrate that TRF1 and TRF2 without the basic domain (TRF2ΔB) suppressed TERRA binding and did not provide protection against RNase H digestion. This suppression of TERRA binding by TRF1 is in line with previous studies that have suggested a role for TRF1 in preventing aberrant interactions at the telomere, thus maintaining telomere stability (21). The specific influence of (TRF2ΔB) mutant highlights the significance of the basic domain in TRF2’s interaction with TERRA and its protective role.

The role of RAD51, an essential protein involved in homologous recombination and DNA repair, TERRA interactions, and telomere stability, has been illuminated by a previous study (10). Our findings extend this understanding by demonstrating that RAD51 engages with TERRA to enhance its annealing to the telomeric duplex to form a triplex structure and to provide partial protection against RNase H degradation. Our EMSA results indicate a strong affinity of RAD51 for TERRA compared to its DNA counterpart, in agreement with previous reports (10). We uncovered that RAD51 prefers binding structured (G4) RNA compared to stretched-out RNA of the same length. Interestingly, the binding of RAD51 does not unfold the G4 structure, as revealed by comparing RAD51 binding to a G4 DNA with and without 8oxoG lesion. RAD51 does not significantly increase the DNA binding to telomeric duplex and triplex formation. Collectively, our finding shows RAD51 increases TERRA engagement and triplex formation without destabilizing the G4 structure, and it can partially protect TERRA from degradation by RNase H in the triplex structure.

Our study broadens the understanding of the intricate mechanisms critical for telomere integrity and stability. The identification of a stable triplex structure, formed during the sequence-specific *trans* association of TERRA with the telomeric duplex, unveils a novel aspect of the protective mechanisms safeguarding against telomere destabilization. Additionally, the role of sub-telomeric sequences, the G4 overhang structure, and modulation by specific proteins adds depth to our comprehension of the complexities involved in telomere maintenance. These findings enrich our existing knowledge base of telomere dynamics and could provide valuable direction for future research in this field.

## Supplementary Material

gkae732_Supplemental_File

## Data Availability

Custom codes are available on GitHub (https://github.com/Ha-SingleMoleculeLab) and archived in Zenodo with the following DOIs: Data acquisition https://doi.org/10.5281/zenodo.4925630, Raw data analysis https://doi.org/10.5281/zenodo.4925617. All raw data is available and can be released upon request from the corresponding author, sua.myong@childrens.harvard.edu.
