# Supplementary material for: The molecular mechanism for TERRA recruitment and annealing to telomeres
Source: Nucleic Acids Res. 2024 Aug 27;52(17):10490–503. doi: 10.1093/nar/gkae732 (PMC11417404; doi:10.1093/nar/gkae732)
Supplement: gkae732_Supplemental_File [file gkae732_supplemental_file.docx]

**SUPPLEMENTARY INFORMATION**

**The Molecular mechanism for TERRA recruitment and annealing to telomeres**

Bersabel Wondimagegnhu^1,2^, Wen Ma^3^,Tapas Paul^2^, Ting-Wei Liao^2,4^, Chun Ying Lee^2^, Samantha Sanford^5,6^, Patricia L Opresko^5,6^, Sua Myong^1,2,4,*^

^1^Program in Cell, Molecular, Developmental Biology and Biophysics, Johns Hopkins University, Baltimore, MD

21218, USA

^2^Program in Cellular and Molecular Medicine, Boston Children’s Hospital, Harvard Medical School, Boston, MA 02115, USA.

^3^Department of Physics, The University of Vermont, Burlington, VT 05405, USA.

^4^Department of Biophysics, Johns Hopkins University, Baltimore, MD 21218, USA

^5^Department of Environmental and Occupational Health, University of Pittsburgh School of Public Health,

Pittsburgh, PA 15261, USA

^6^UPMC Hillman Cancer Center, Pittsburgh, PA, 15213

* To whom correspondence should be addressed: [sua.myong@childrens.harvard.edu](mailto:sua.myong@childrens.harvard.edu).

Supplementary information includes:

Supplementary Table 1 and Table 2

Supplementary Figures, S1-S5

**Supplementary Table 1.** DNA and RNA Oligonucleotides (5’ to 3’) used in these experiments.

|  | RNA Samples | Sequences and modifications |
| --- | --- | --- |
| 1 | 18_TERRA[G4] _Cy3 | rUrGrGrCrGrArCrGrGrCrArGrCrGrArGrGrCrUrUrArGrGrGrUrUrArGrGrGrUrUrArGrGrGrUrUrArGrGrG/Cy3/ |
| 2 | TERRA[G4] _Cy3 | rUrUrArGrGrGrUrUrArGrGrGrUrUrArGrGrGrUrUrArGrGrG /Cy3/ |
| 3 | 18RNA_Cy3 | rUrGrGrCrGrArCrGrGrCrArGrCrGrArGrGrC /Cy3/ |
| 4 | Poly U (18) _TERRA[G4]_Cy3 | rUrUrUrUrUrUrUrUrUrUrUrUrUrUrUrUrUrUrUrUrArGrGrGrUrUrArGrGrGrUrUrArGrGrGrUrUrArGrGrG /Cy3/ |
| 5 | Cy3-TERRA[G4] | /Cy3/rUrUrArGrGrGrUrUrArGrGrGrUrUrArGrGrGrUrUrArGrGrG |
| 6 | 15Q18-TERRA | rCrCrCrArArArArUrArUrArArCrArCrCrCrArUrUrArGrGrGrUrUrArGrGrGrUrUrArGrGrGrUrUrArGrGrG/Cy3/ |
| 7 | Cy5_TERRA[G4] _Cy3 | /Cy5/UrUrArGrGrGrUrUrArGrGrGrUrUrArGrGrGrUrUrArGrGrG/Cy3/ |
| 8 | Poly U (40)_Cy3 | rUrUrUrUrUrUrUrUrUrUrUrUrUrUrUrUrUrUrUrUrUrUrUrUrUrUrUrUrUrUrUrUrUrUrUrUrUrUrUrU/Cy3/ |
|  |  |  |
|  | DNA Samples | Sequences and modifications |
| 1 | Telomere duplex (C-rich Cy5 and biotinylated strand) | CCCTCy5AACCCTAACCCTAACCCTAAGCCTCGCTGCCGTCGCCA-biotin |
| 2 | Telomere duplex (G-rich unbiotinylated strand) | TGGCGACGGCAGCGAGGCTTAGGGTTAGGGTTAGGGTTAGGG |
| 3 | 18_DNA[G4] _Cy3 | TGGCGACGGCAGCGAGGCTTAGGGTTAGGGTTAGGGTTAGGG-/Cy3/ |
| 4 | DNA[G4] _Cy3 | TTAGGGTTAGGGTTAGGGTTAGGG/Cy3/ |
| 5 | 18DNA_Cy3 | TGGCGACGGCAGCGAGGC/Cy3/ |
| 6 | Telomere duplex -15Q 18-G4-DNA (G-rich unbiotinylated strand) | CCCAAAATATAACACCCA TTAGGG TTAGGGTTAGGGTTAGGG |
| 7 | Telomere duplex 15Q 18-Crich-DNA (Cy5 and biotinylated strand) | CCCTCy5AACCCTAACCCTAACCCTAATGGGTGTTATATTTTGGG-biotin |
| 8 | 18_Cy3DNA[G4] _Cy5 | TGGCGACGGCAGCGAGGC/Cy3/TTAGGGTTAGGGTTAGGGTTAGGG/Cy5/ |
| 9 | 18-DNA[G4]-T24 | TGGCGACGGCAGCGAGGCTTAGGGTTAGGGTTAGGGTTAGGGTTTTTTTTTTTTTTTTTTTTTTTT |
| 10 | 18-DNA[G6] | TGGCGACGGCAGCGAGGCTTAGGGTTAGGGTTAGGGTTAGGGTTAGGGTTAGGG |
| 11 | 18-DNA[G7] | TGGCGACGGCAGCGAGGCTTAGGGTTAGGGTTAGGGTTAGGGTTAGGGTTAGGGTTAGGG |
| 12 | 18-DNA[G8] | TGGCGACGGCAGCGAGGCTTAGGGTTAGGGTTAGGGTTAGGGTTAGGGTTAGGGTTAGGGTTAGGG |
| 13 | 18-G4-G2r2-8oxoG | TGGCGACGGCAGCGAGGCTTAGGGTTAG (8oxoG)GTTAGGGTTAGGG |
| 14 | TERRA transcription top strand | TGGCGACGGCAGCGAGGCTAAATTAATACGACTCACTATAGGGAGACCACAACG/iCy3/TAGGGTTAGGGTTAGGGTTAGG GT/Cy5/ATCAGCTCCAGGTCT |
| 15 | TERRA transcription bottom strand | AGACCTGGAGCTGATAACCCTAACCCTAACCCTAACCCAACGTTGTGGTCTCCCTATAGTGAGTCGTATTAATTTA |
| 16 | Biotin-18 mer | GCCTCGCTGCCGTCGCCA-Biotin |

**Supplementary Table 2.** Maximum and kd Values for binding

|  | **Max** | **kd** |  |  | **Max** | **kd** |
| --- | --- | --- | --- | --- | --- | --- |
| **Figure 1C** |  |  |  | **Figure 4D** |  |  |
| TERRA-18 | 0.529 | 4.383 |  | With 8oxoG | 0.679 | 4.012 |
| ss18(RNA) | 0.219 | 2.811 |  | Without 8oxoG | 0.529 | 4.383 |
| TERRA | 0.182 | 21.768 |  | **Figure 5B** |  |  |
| ss40 | 0 | 0 |  | TRF2 | 0.505 | 0.071 |
| **Figure 1D** |  |  |  | None | 0.529 | 4.383 |
| G4-18 | 0.69 | 6.339 |  | TRF1 | 0.28 | 0.294 |
| G4 | 0.469 | 3.651 |  | TRF2ΔB | 0.197 | 4.717 |
| ss18(DNA) | 0.182 | 1.198 |  | **Figure 6C** |  |  |
| **Figure 1F** |  |  |  | withRAD51 | 0.902 | 2.39 |
| TERRA-15q(18) | 0.475 | 4.59 |  | withoutRAD51 | 0.495 | 3.166 |
| **Figure 4B** |  |  |  | **Figure 6E** |  |  |
| G4-POT1 | 1.06 | 6.969 |  | withRAD51 | 0.6 | 1.427 |
| G4 | 0.983 | 6.151 |  | withoutRAD51 | 0.468 | 0.2572 |
| G3 | 0.521 | 2.45 |  |  |  |  |
| G2 | 0.489 | 5.34 |  | **Figure 1 supplement** |  |  |
| T24 | 0.482 | 4.765 |  | TERRA - Poly U (18) | 0.337 | 4.789 |
| No overhang | 0.529 | 4.383 |  |  |  |  |
| **Figure 4C** |  |  |  |  |  |  |
| G4 | 0.577 | 2.906 |  |  |  |  |
| G3 | 0.175 | 2.153 |  |  |  |  |
| G2 | 0.192 | 4.532 |  |  |  |  |
| T24 | 0.336 | 6.05 |  |  |  |  |
| No overhang | 0.182 | 21.768 |  |  |  |  |

**
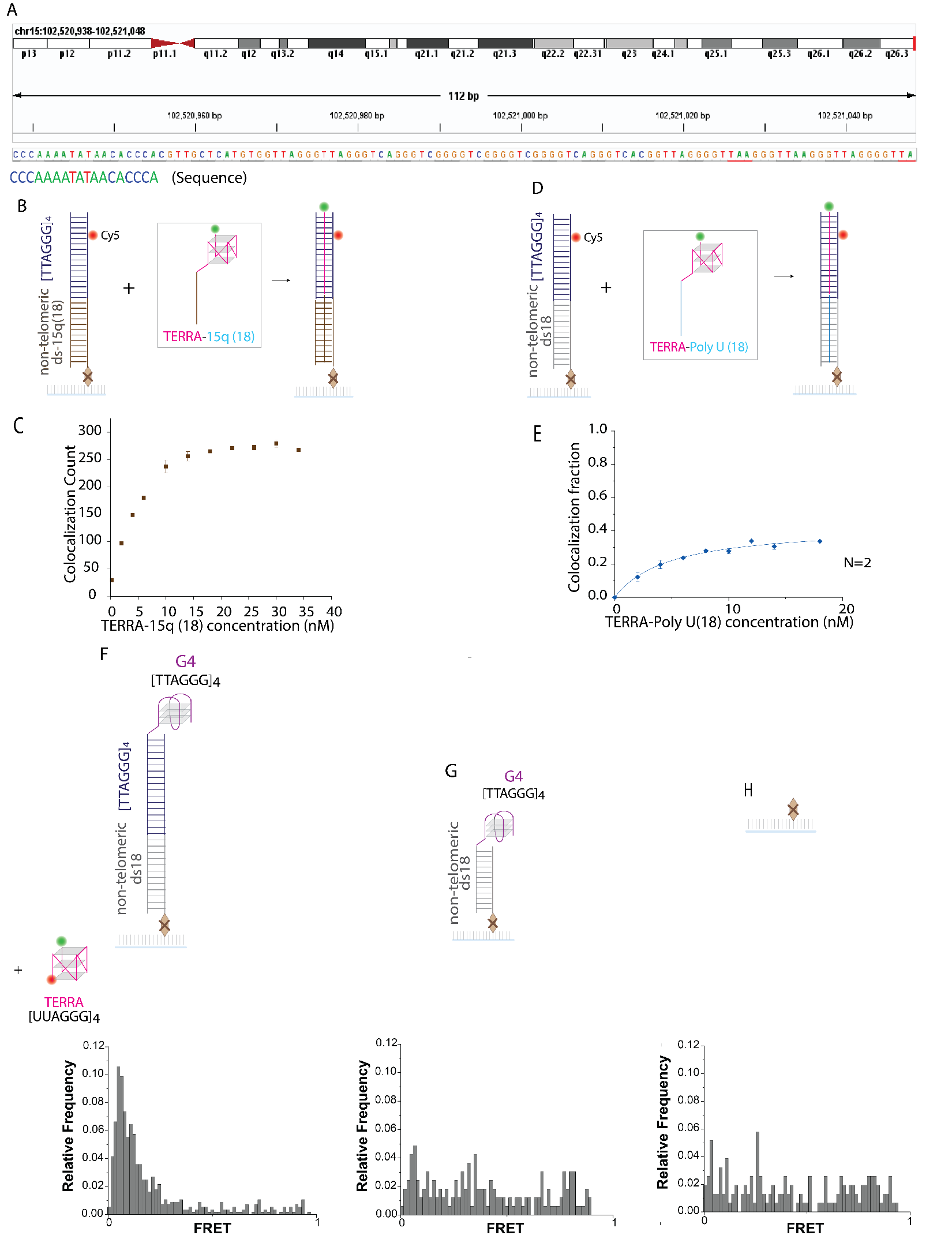
**

**Figure S1:** **TERRA *trans* annealing to telomeric duplex DNA is enhanced by sub-telomeric sequence. (A)** The location of the 15q (18) on chromosome 15 immediately preceding the telomere sequence. **(B &C)** The binding of 15q (18)-TERRA to telomeric duplex with 15q (18) base and colocalization count. **(D)** DNA construct: Cy5 labeled telomeric duplex [TTAGGG]_4_ with 18 bp located adjacent to telomeric duplex. PolyU-TERRA-Cy3 in which TERRA RNA consisted of [UUAGGG]_4_) and Poly U is an 18 Uracil nucleotides is applied to the immobilized DNA. **(E)** Poly U (18) – TERRA binding to a telomeric duplex with 18mer base. **(F, G, H)** Histogram for FRET distribution upon applying Cy5 TERRA [UUAGGG]_4_ -Cy3 to telomeric duplex with 18mer base and a G4 overhang, 18mer base or to surface respectively.

**
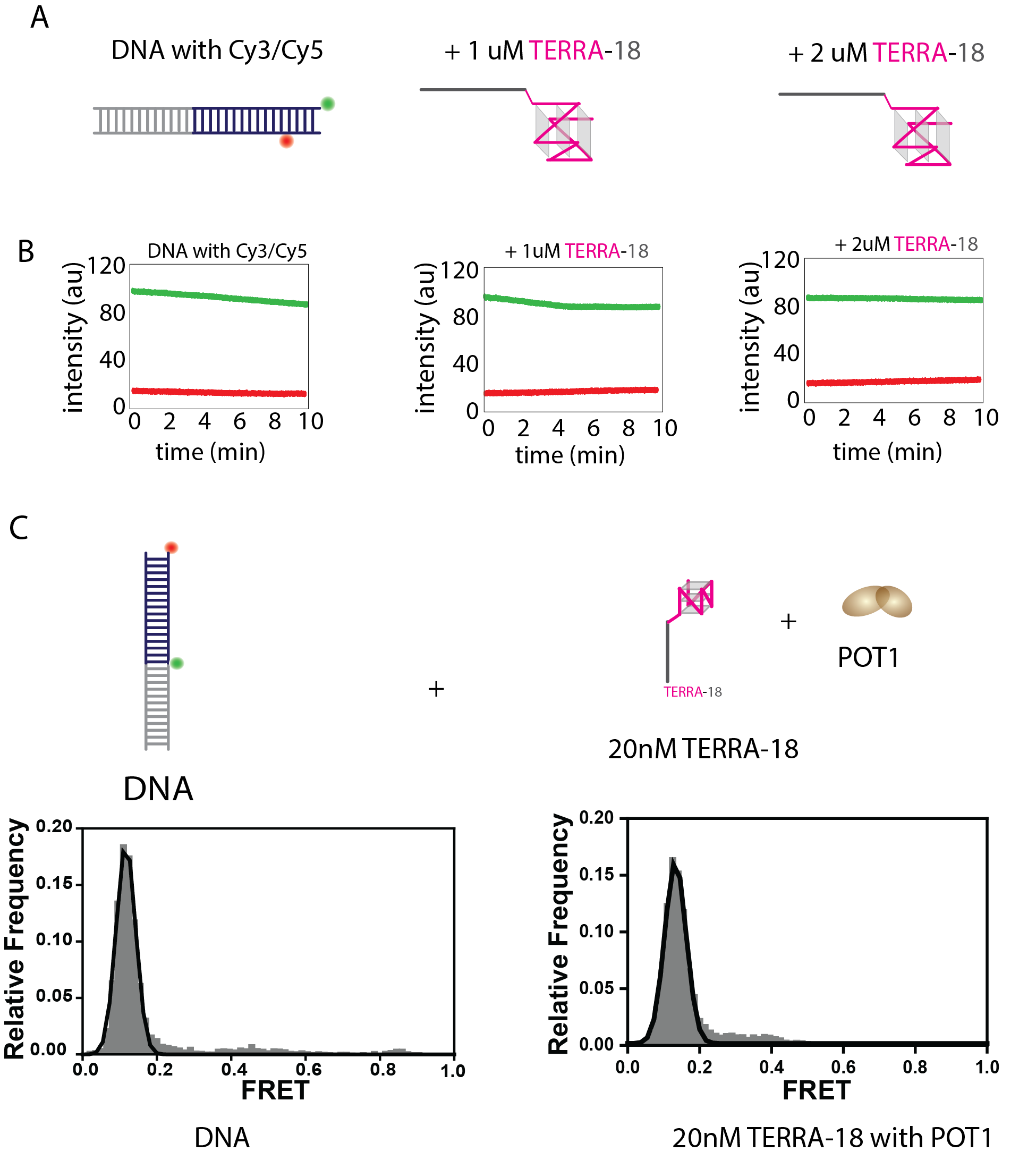
**

**Figure S3:** **TERRA does not displace the G-rich strand.**

**(A&B)** Spectrofluorometer measurements of Cy3 and Cy5 fluorescence intensity before and after the addition of unlabeled 18- TERRA (1μM and 2μM) to a telomeric DNA labeled with Cy3 and Cy5 remains the same. **(C)** Low FRET state is maintained upon the addition of excess unlabeled TERRA + POT1 to a telomeric duplex with the G-rich strand labeled with Cy3 and Cy5 (Cy5- [TTAGGG]_4_ -Cy3).


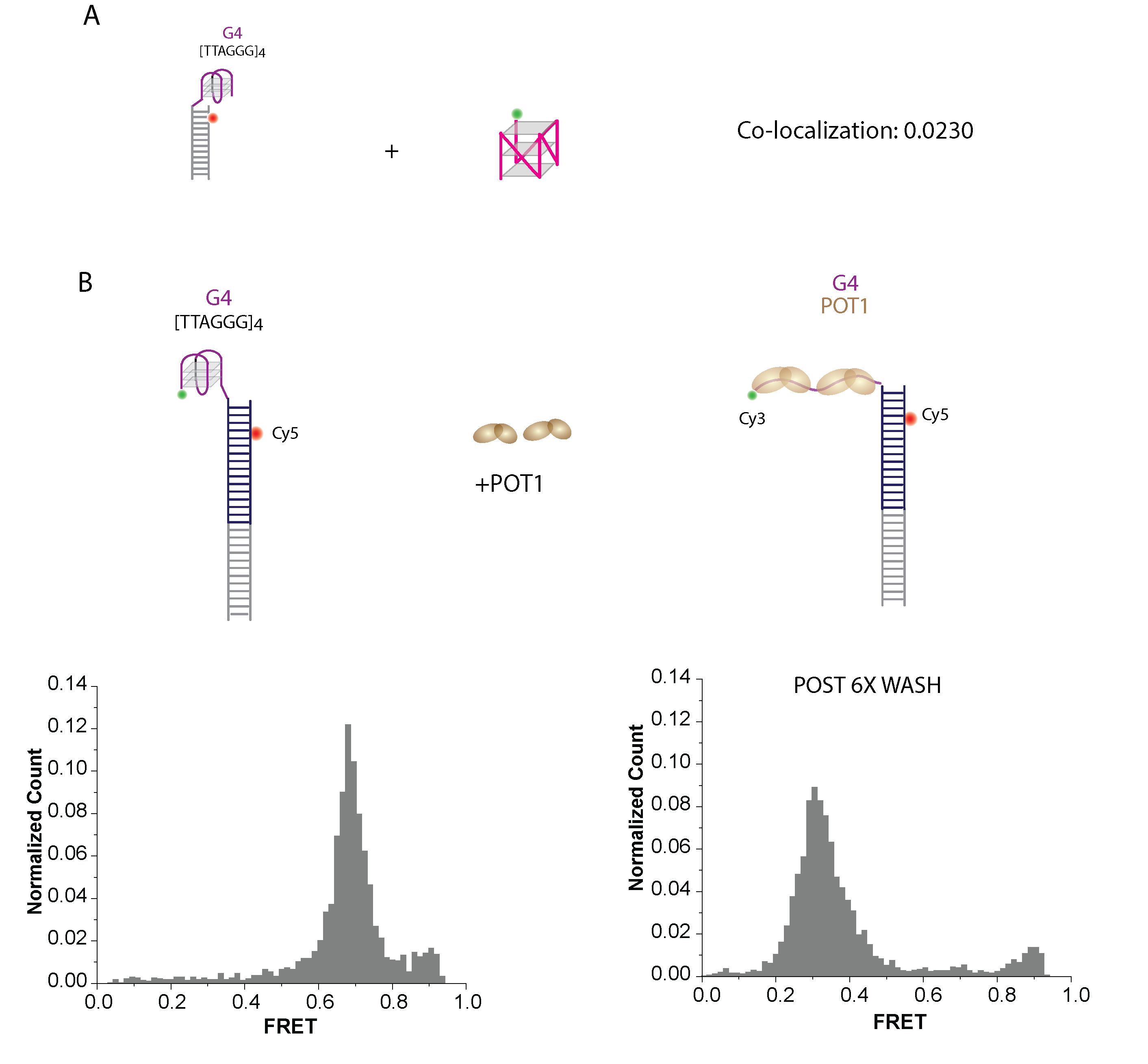


**Figure S4:** **(A)**18mer-G4 DNA colocalization with TERRA (up to 12nM) **(B)** POT1 stably binds to G4 overhang post 6x washes.

**
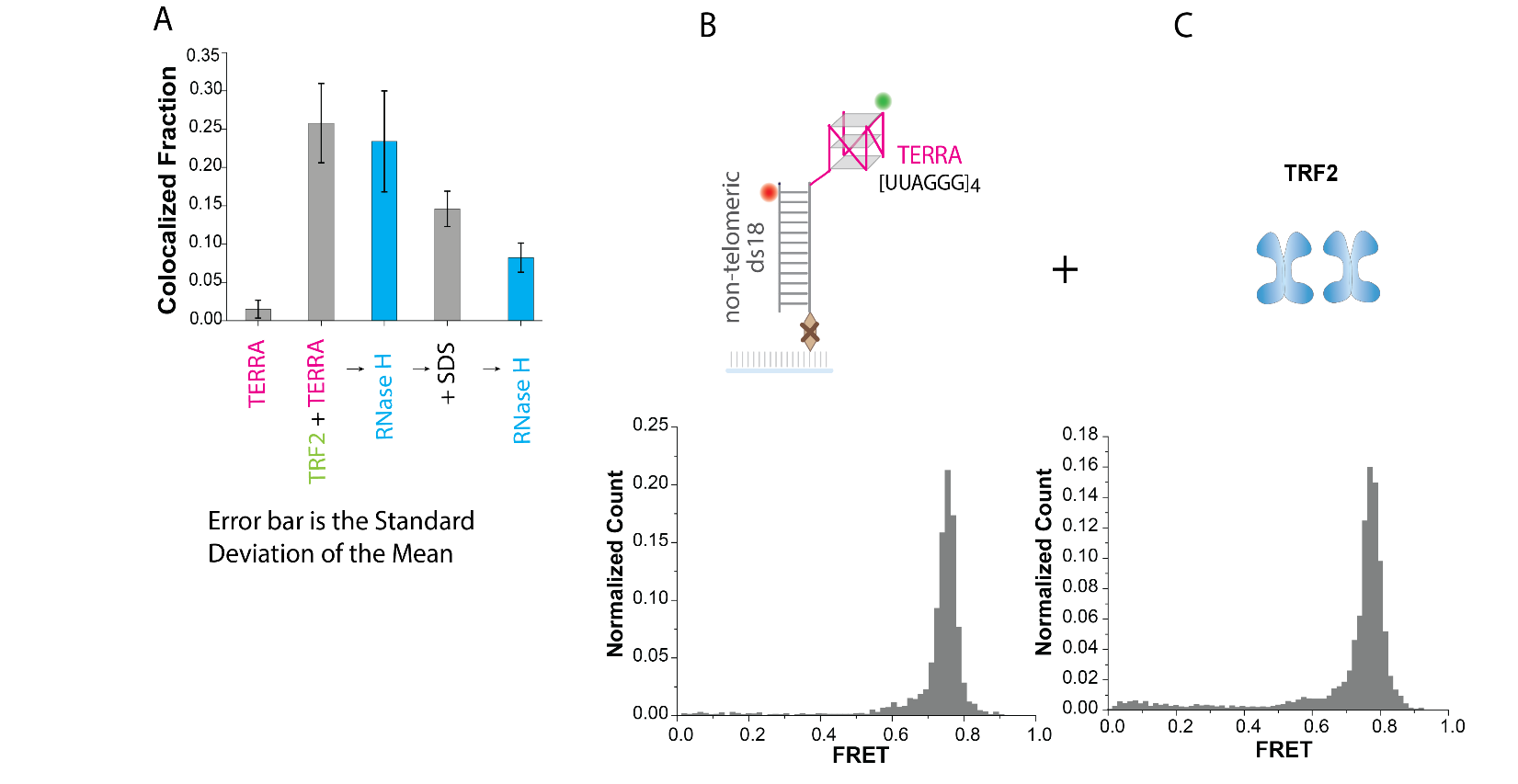
**

**Figure S5:** **TRF2 promotes TERRA binding and protects TERRA from RNase H digestion. (A)** TERRA (0.25nM) and telomere DNA colocalization with and without TRF2 and RNase H treatment pre and post SDS denaturation of TRF2. **(B-C)** FRET histogram of TERRA before and after the addition of TRF2.
